# Exploring How AI Literacy and Self-Regulated Learning Relate to Student Writing Performance and Well-Being in Generative AI-Supported Higher Education

**DOI:** 10.3390/bs15050705

**Published:** 2025-05-20

**Authors:** Jiajia Shi, Weitong Liu, Ke Hu

**Affiliations:** 1 School of International Education, Shandong University, Jinan 250100, China; shi@mail.sdu.edu.cn; 2 Institute for Advanced Studies in Education, Shandong University, Jinan 250100, China; 3 School of Education, Fujian Normal University, Fuzhou 350007, China

**Keywords:** generative artificial intelligence, AI literacy, self-regulated learning, well-being, writing performance, higher education

## Abstract

The integration of generative artificial intelligence (GAI) into higher education is transforming students’ learning processes, academic performance, and psychological well-being. Despite the increasing adoption of GAI tools, the mechanisms through which students’ AI literacy and self-regulated learning (SRL) relate to their academic and emotional experiences remain underexplored. This study investigates how AI literacy and SRL are associated with writing performance and digital well-being among university students in GAI-supported higher learning contexts. A survey was administered to 257 students from universities in China, and structural equation modeling was used to examine the hypothesized relationships. Results show that both AI literacy and SRL significantly and positively predict students’ writing performance, with SRL having a stronger effect. Moreover, AI literacy shows a positive association with GAI-driven well-being, with writing performance serving as a partial mediator in this relationship. These findings suggest that fostering both technological competencies and effective learning strategies may support students’ academic outcomes while supporting their psychological well-being in AI-enriched educational environments. By integrating AI literacy and SRL into a unified model, this study contributes to the growing body of research on GAI-driven well-being in higher education and offers practical implications for cultivating balanced and sustainable learning experiences in the age of GAI.

## 1. Introduction

The rapid development of artificial intelligence (AI) is reshaping the relationship between individual learners and academic performance in higher education. With the emergence of generative AI (GAI) tools such as ChatGPT (GPT-4) and DeepSeek (DeepSeek-V3), researchers have begun exploring their use in academic writing. Recent studies show that GAI can improve writing quality by enhancing grammar, vocabulary, and fluency. It can also save time and offer personalized feedback and automated evaluation (Lund & Wang, 2023; Chen, 2023; Salvagno et al., 2023; Zawacki-Richter et al., 2019).

Beyond academic performance, researchers have also emphasized the psychological impact of GAI use, especially its effect on student well-being. Well-being is broadly defined as an individual’s positive subjective experience and evaluation of life (Tov, 2018), encompassing dimensions such as self-regard, mastery of the surrounding environment, quality relations with others, continued growth and development, purposeful living, and so on (Ryff & Keyes, 1995). In digital contexts, it includes users’ sense of control, comfort, and satisfaction with technology (Burr et al., 2020; Vanden Abeele, 2021; Gui et al., 2017). Recent research further suggests that interactions with GAI tools themselves—especially in emotionally expressive or supportive contexts—may also contribute directly to improved affective states (Hu et al., 2025), expanding the potential impact of GAI beyond academic outcomes.

This study focuses on GAI-driven well-being, which refers to students’ overall emotional, social, and psychological state while using GAI for learning. Unlike brief digital satisfaction, GAI-driven well-being reflects a sustained positive experience shaped through interaction with AI tools in academic settings. Prior research has identified potential benefits such as personalized learning, reduced stress, and improved self-awareness (Shahzad et al., 2024; Makhambetova et al., 2021). However, overdependence on GAI may also lead to digital fatigue, loneliness, anxiety, and technostress (Cambra-Fierro et al., 2025; Crawford et al., 2024; Klimova & Pikhart, 2025).

Researchers are increasingly interested in how both technology and learner characteristics shape academic and psychological outcomes. In writing contexts, one of the most influential individual factors is SRL—referring to the behavior and process in which learners acquire information or skills through perception of subjectivity, purposefulness, and instrumentality (Zimmerman, 1990; Zimmerman & Schunk, 2001; Teng & Zhang, 2020; Yang et al., 2023). At the same time, in digital and intelligent learning environments, AI literacy has emerged as a critical personal attribute linked to students’ ability to effectively use GAI tools for academic writing and better well-being. With the development of times, AI literacy has evolved from merely acquiring knowledge and skills about AI (Kandlhofer et al., 2016) to a multidimensional construct encompassing technical proficiency, attitudes, emotional responses, values, and ethical awareness related to AI use (Ng et al., 2021; C. R. Wang & Wang, 2025).

Although past research has examined the link between student traits, performance, and well-being in digital environments, a few studies have built a model integrating all these factors. On the one hand, few studies have focused on the overall impact of AI literacy and SRL on writing performance. On the other hand, although many scholars have studied the effects of these two factors on user happiness separately, no in-depth investigation has been conducted on writing in a generative GAI environment. This study addresses that gap by examining how AI literacy and SRL jointly relate to students’ writing performance and well-being. In the context of GAI-driven changes to higher education, it is important to understand students not only as users of technology but as cognitive, emotional, and behavioral agents. This research contributes a new perspective on how learner skills and emotional states are connected in the era of GAI.

## 2. Literature Review

### 2.1. The Influence of Learner Factors in GAI-Supported Writing Environments

Learners’ ability to effectively apply GAI tools in writing tasks is shaped not only by technological affordances but also by individual-level psychological and cognitive factors. Among these, AI literacy has emerged as a critical construct in recent research, reflecting a learner’s readiness and capacity to engage meaningfully with AI in educational settings. The term has expanded its conceptual scope. For instance, Long and Magerko (2020) defined AI literacy as a set of competencies that enables individuals to critically evaluate AI technologies, communicate and collaborate effectively with AI, and use AI as a tool online, at home, and in the workplace. Ng et al. (2021) proposed a widely cited four-dimensional framework that includes the following: (1) knowing and understanding the basic concepts and functionalities of AI, (2) using and applying AI applications in various contexts, (3) evaluating and creating AI tools, and (4) being ethical and socially responsible users. Building on this foundation, Warschauer et al. (2023) tailored a five-dimensional model for second language (L2) writers, while C. R. Wang and Wang (2025) proposed the APSE (A: critical awareness of AI, P: critical positionality, S: critical strategies for interacting with AI, and E: critical evaluation of AI affordances) model, which systematically elucidates the dynamic linkages between AI literacy and writing performance in GAI-supported contexts.

Recent empirical studies further suggest that learners with advanced prompt engineering and critical questioning skills can substantially improve both the quality and efficiency of GAI-supported content while also mitigating the risks of factual hallucinations—a phenomenon where large language models fabricate inaccurate information that appears plausible and convincing to users (Onder & McCabe, 2025; Slater & Humphries, 2025). These hallucinations vary in severity, ranging from subtle deviations from established facts to completely fictional or nonsensical statements (Maleki et al., 2024). These findings have led to the development of extended models of AI literacy that incorporate elements such as critical inquiry ability (Mzwri & Turcsányi-Szabo, 2025). Such theoretical progress marks an important shift in the understanding of AI literacy—from a narrow focus on technical proficiency to a broader, integrated cognitive framework that includes metacognition, critical thinking, and ethical reasoning. This evolution also signals a corresponding shift in assessment paradigms from static, knowledge-based evaluations to dynamic, process-oriented analyses that better reflect learners’ ability to interact meaningfully and responsibly with GAI tools.

In parallel, SRL theory provides a complementary perspective for understanding how learners engage with GAI feedback to refine their writing strategies. Grounded in Bandura’s (1991) model of triadic reciprocal causation—which emphasizes the interaction between personal, behavioral, and environmental determinants—SRL is defined as a cyclical process involving forethought (planning), performance (implementation), and self-reflection (monitoring and adjustment) (Zimmerman & Schunk, 2001). This framework has been extended to digital learning environments by Barnard et al. (2009), who developed an SRL model specific to online contexts. Subsequent advancements in learning analytics, such as the behavioral analysis techniques introduced by D. Kim et al. (2018), have further enabled researchers to explore SRL processes in technologically mediated writing environments. Empirical studies have shown that targeted SRL interventions can significantly enhance students’ writing performance (Bai, 2015; De Silva & Graham, 2015). Moreover, SRL has been found to indirectly improve writing outcomes by strengthening learners’ language-specific self-efficacy (Teng & Zhang, 2020). More recently, Shen and Bai (2024) proposed an integrated model that explicitly aligns stages of the writing process—planning, drafting, and revising—with corresponding SRL phases. This alignment offers a valuable theoretical lens for analyzing how learners cognitively regulate their engagement with GAI throughout different stages of composition.

Although prior research has separately confirmed the predictive power of AI literacy and SRL on writing outcomes, few studies have examined their synergistic effects within GAI-supported writing contexts. That is, while both constructs have been recognized as important individually, the potential for SRL and AI literacy to jointly influence writing performance has remained underexplored—particularly in digitally mediated academic settings where learners must navigate complex cognitive, technical, and ethical demands.

### 2.2. The Opportunities and Challenges of GAI in Academic Writing

Breakthroughs in natural language processing technologies have triggered a paradigmatic shift in academic writing practices. GAI tools have become deeply embedded across the academic writing workflow, supporting tasks ranging from research question formulation and literature review assistance to code validation and grant proposal drafting (Yan et al., 2024). A growing body of research has confirmed that GAI can enhance the writing process across multiple dimensions while also offering emotional and cognitive support to learners (Lund & Wang, 2023; J. Kim et al., 2025). At the task-execution level, GAI enables end-to-end assistance—from ideation and planning to language refinement, editing, and revision—and provides contextual background for writing topics, often adapting to users’ search preferences and content needs (Rowland, 2023). In terms of quality assurance, AI facilitates error detection and consistency, identifying grammatical mistakes (Fitria, 2021), thereby improving textual accuracy and coherence (Liu et al., 2024). For cross-lingual writing, GAI enables students to overcome language barriers to access and assimilate content in multiple languages and learn diverse perspectives (Salvagno et al., 2023). Importantly, GAI not only assists with low-level linguistic edits but also contributes to high-order cognitive processes, such as brainstorming, critical feedback, and creative writing support (Z. Lin, 2024).

Nevertheless, the enhancement of efficiency through GAI brings with it deeper challenges related to learners’ development and academic performance. Existing research has primarily focused on three areas of concern: the reliability of GAI outputs, the potential impact on learners’ cognitive skills, and academic integrity issues (J. Kim et al., 2025). A major challenge is the phenomenon of hallucination, wherein GAI generates superficially plausible but factually incorrect information (Alkaissi & McFarlane, 2023). Fyfe (2023) found that 87% of learners working with AI-assisted tools reported increased cognitive load due to the need for extensive verification of AI-generated content. Bašić et al. (2023) reported inconclusive results from quasi-experimental studies, indicating that AI-assisted groups often felt uncertain about the usefulness of the tools, and in some cases, doubted the overall efficacy of GAI in writing (Choudhuri et al., 2024).

Moreover, the simplification of information search and answer generation facilitated by GAI may inadvertently undermine students’ critical thinking and problem-solving abilities. Long-term use could lead to the erosion of independent reasoning and higher-order cognitive skills (Kasneci et al., 2023). Beyond cognitive and academic outcomes, ethical concerns have also been raised. Scholars warn that uncritical adoption of AI-generated suggestions may increase the likelihood of both intentional and unintentional plagiarism, as well as passive overreliance on AI input (Prentice & Kinden, 2018; Rogerson & McCarthy, 2017; Salvagno et al., 2023). These issues raise broader questions about academic ethics and the future of scholarly integrity (Miao & Holmes, 2023; Memarian & Doleck, 2023).

Currently, the growing body of research on the benefits and limitations of GAI in academic writing highlights the importance of not only asking how GAI affects learners but also exploring the reverse mechanism—namely, how learners’ own competencies and performance influence the effectiveness of GAI tool use. Learner characteristics and GAI interactions should therefore be viewed as part of a complex system. To meaningfully support learners in achieving successful transfer and sustainable development within GAI-supported writing environments, it is essential to conduct more systematic and holistic investigations into this mechanism.

### 2.3. The Impact of GAI on Student Well-Being in Higher Education

As a technological mediator, GAI has drawn increasing scholarly attention for its profound impact on student learning in higher education—particularly regarding student well-being. Existing research suggests that this impact is fundamentally dual in nature: while GAI can reduce instructional pressure, increase engagement, empathy development, and promotion of well-being (Sethi & Jain, 2024) through personalized support, it may also pose risks to students’ psychological well-being due to technology dependency, ethical concerns, and reduced social connectedness (Crawford et al., 2024).

The positive effects of GAI on student well-being have been highlighted in at least three key areas. First, GAI can dynamically assess learning needs and adapt instructional content accordingly, providing students with targeted support that enhances both academic performance and emotional well-being. Second, GAI facilitates the creation of more equitable and inclusive learning environments, helping to reduce anxiety and feelings of isolation commonly experienced by students with unique learning challenges (Flavian & Alstete, 2024; Zhang et al., 2025). Third, GAI has been found to increase student engagement and motivation—ultimately enhancing their sense of learning achievement, self-efficacy, and well-being (Shahzad et al., 2024).

Nevertheless, the role of GAI as a technological mediator may also exert adverse effects on students’ well-being. Studies have shown that excessive reliance on GAI reduces the frequency of face-to-face interactions. This is especially concerning in entertainment-like application scenarios, where real-world social skills tend to deteriorate. As a result, students may become more socially isolated and less capable of engaging in in-person communication and collaboration, factors that are crucial to their broader social well-being and developmental outcomes (Cambra-Fierro et al., 2025; Rodway & Schepman, 2023; Zhai et al., 2024). In addition, Crawford et al. (2024) found that when students perceive GAI as their primary source of academic support, their feelings of loneliness may increase. Supporting this finding, Xie et al. (2023) employed a mixed-methods approach and confirmed a significant association between increased GAI use and heightened levels of social isolation. Moreover, students in higher education may experience growing levels of technological anxiety as their dependence on GAI tools increases, particularly when they lack sufficient training or AI-related competencies (González-López et al., 2021). The expanding use of GAI in educational contexts has also raised concerns regarding data privacy and security. GAI systems typically require access to large volumes of student data, including academic records and even personal information (Akgun & Greenhow, 2022). While such data are often leveraged to optimize learning outcomes, they also present risks of misuse or privacy breaches, potentially compromising students’ psychological and emotional well-being (Malik et al., 2024).

Taken together, these concerns point to the urgent need for continued research on how to support student well-being in GAI-supported learning environments. Identifying the key factors that influence learners’ well-being—and developing strategies to strengthen it—remains a critical area of inquiry as GAI becomes increasingly integrated into higher education.

### 2.4. The Present Study and Hypothetical Model

The integration of GAI into higher education has brought new opportunities and challenges to student learning and psychological development. Prior research has established that GAI can enhance academic writing performance by improving language complexity, accuracy, and fluency while also supporting personalized learning experiences. At the same time, concerns have emerged regarding the risks of overreliance on GAI, including reduced social engagement, increased loneliness, technological anxiety, and potential threats to data privacy—all of which may negatively impact student well-being.

In exploring the impact of technology on student well-being, both the Technology Acceptance Model (TAM) and the Control-Value Theory (CVT) emphasize the interaction between individual and environmental factors. However, these two theoretical frameworks differ in their focus as follows: TAM highlights the instrumental role of cognitive processing, positing that users’ behavioral intentions to adopt technology are driven by their subjective evaluations of the system’s characteristics—namely, perceived usefulness and perceived ease of use—thus following a rational, utility-based decision-making process (Davis, 1989; Davis et al., 1989; Silva, 2015). In contrast, CVT asserts that achievement emotions stem from individuals’ subjective appraisals of task control and value (Pekrun, 2006; Pekrun et al., 2007; Pekrun et al., 2014). In this framework, decisions are primarily shaped by emotional feedback, emphasizing the affective responses to the learning experience.

In this evolving learning context, individual learner characteristics have become increasingly salient. Specifically, AI literacy and SRL have been identified as two critical competencies that enable students to effectively engage with GAI tools. However, while both AI literacy and SRL have been independently linked to academic outcomes, a few studies have examined their combined effects—particularly on writing performance and GAI-driven well-being. Moreover, existing research tends to treat these variables in isolation or through linear models, with limited attention to how they might function synergistically in AI-enhanced learning processes. Although AI literacy reflects students’ technological competencies and SRL reflects their strategic learning behaviors, they may together relate to not only cognitive outcomes such as performance but also affective outcomes such as digital well-being. To date, the interrelationships among AI literacy, SRL, writing performance, and GAI-driven well-being have not been systematically examined. To address these gaps, the present study develops and tests a conceptual model that integrates AI literacy and SRL to investigate their joint influence on university students’ writing performance and digital well-being in GAI-supported academic settings. Drawing on theories of SRL (Zimmerman & Schunk, 2001), AI literacy (Ng et al., 2021), and the dialectical impacts of GAI on well-being (Shahzad et al., 2024; Cambra-Fierro et al., 2025), this study proposes a mediated model in which writing performance serves as a mechanism through which learner competencies influence psychological outcomes.

**Figure 1:**
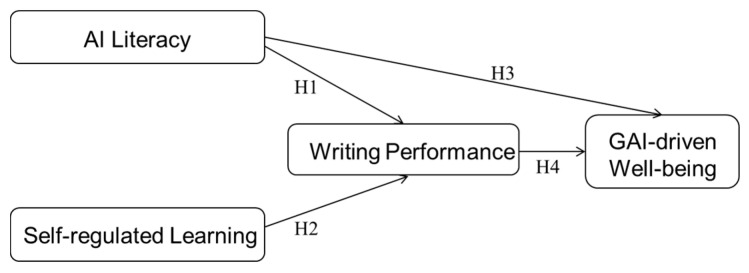
Hypothetical Model.

As illustrated in the hypothetical model (see Figure 1), this study contributes to a systems-level understanding of learner development, conceptualizing it as an integrated process encompassing cognitive, behavioral, and emotional dimensions within technology-enhanced environments. Based on the proposed model, the following hypotheses are formulated:

**H1.**  *AI literacy is positively associated with writing performance*.

**H2.**  *Self-regulated learning is positively associated with writing performance*.

**H3.**  *AI literacy is positively associated with GAI-driven well-being*.

**H4.**  *Writing performance is positively associated with GAI-driven well-being*.

By exploring these questions, this study aims to contribute to a more integrated understanding of how cognitive, behavioral, and emotional dimensions interact within GAI-enhanced learning environments. The findings are expected to inform educational design, instructional support, and policy development aimed at promoting both academic success and psychological flourishing in the age of artificial intelligence.

## 3. Materials and Methods

### 3.1. Participants and Procedure

Participants in this study were undergraduate and graduate students from a high-level comprehensive university located in northern China. A total of 275 students initially participated in the survey. After excluding incomplete responses, 257 valid questionnaires were retained for analysis. Among the valid respondents, 89 (34.6%) were male and 168 (65.3%) were female. In terms of academic level, 12 participants (4.6%) were freshmen, 47 (18.2%) sophomores, 74 (28.7%) juniors, and 51 (19.8%) seniors. The sample also included 43 (16.7%) first-year graduate students, 24 (9.3%) second-year graduate students, and 6 (2.3%) third-year graduate students. In terms of nationality, 224 students (87.1%) were from China, while 33 (12.8%) were international students. Participants came from a variety of academic disciplines, including humanities and arts (29.6%), social sciences (50.6%), natural sciences (10.9%), and engineering sciences (8.9%), as shown in Table 1.

The questionnaire was designed based on the relevant literature and theoretical frameworks and finalized in November 2024 after two rounds of pilot testing. Given that some instruments were originally developed in English and the target participants were Chinese students, pilot testing was essential to ensure contextual and linguistic appropriateness. One round of the pilot test involved undergraduate students, while the other involved domain experts. Feedback from both groups was used to refine the questionnaire items and improve construct validity. The formal survey was conducted between 12 December and 18 December 2024, using Wenjuanxing (https://www.wjx.cn/, accessed on 11 December 2024), a widely used online survey platform in China. Data were collected over a one-week period using random sampling procedures. To ensure anonymity and minimize any potential risks, the survey was administered online with no identifiable personal information collected. A statement at the beginning of the questionnaire informed participants that their participation was voluntary, that they could skip any question, and that submitting the questionnaire constituted informed consent. Respondents were also informed of their right to withdraw from this study at any point without penalty.

### 3.2. Instruments

The survey instrument was developed based on relevant theories and prior empirical research and consisted of two main sections. The first section collected participants’ demographic information, including gender, age, grade level, and academic discipline. The second section comprised four multi-item scales designed to measure the core constructs in the research model: AI literacy, SRL, writing performance, and GAI-driven well-being (see Appendix A for detailed items).

The AI literacy scale was primarily adapted from the instrument developed by B. Wang et al. (2023), which was designed to capture the AI literacy of general users. This scale covers four core dimensions: awareness, use, evaluation, and ethics. In addition, we consulted related literature by Almatrafi et al. (2024), Druga et al. (2023), and Ng et al. (2021) to further refine the scale items. The internal consistency (Cronbach’s α) for this scale was 0.83, indicating good reliability. The SRL scale was based on Zimmerman and Pons (1986), focusing on key self-regulation strategies such as planning, monitoring, and reflection. SRL items were used to measure students’ application of SRL strategies during GAI-supported learning (Cronbach’s α = 0.75). The writing performance scale was adapted from Bauer and Anderson (2000), who proposed an online assessment rubric with the following three major components: content, expression, and participation. These components were used to evaluate students’ perceived writing performance in digital learning contexts (Cronbach’s α = 0.68). The GAI-driven well-being scale was developed based on the framework proposed by Vanden Abeele (2021), which conceptualizes digital well-being as a dynamic and context-dependent construct. The scale was adapted to reflect the specific context of GAI-supported learning environments (Cronbach’s α = 0.82). All four constructs were measured using 5-point Likert-type scales ranging from 1 (strongly disagree) to 5 (strongly agree). The items were pilot-tested prior to the formal survey and demonstrated satisfactory reliability and construct validity.

### 3.3. Statistical Analyses

This study aims to investigate the mechanisms through which AI literacy, SRL, and writing performance influence GAI-driven well-being in the context of GAI-assisted academic writing. Based on the proposed conceptual model, the hypothesized relationships among the independent, mediating, and dependent variables were derived from both theoretical foundations and prior empirical research. Given the presence of multiple latent constructs and hypothesized pathways, structural equation modeling (SEM) was selected as an appropriate and robust analytical technique (Hair et al., 2014).

The data analysis proceeded in three phases. First, exploratory factor analysis (EFA) was conducted using SPSS (version 22.0) to identify the underlying factor structure and assess the internal reliability of the measurement instruments. The Kaiser–Meyer–Olkin (KMO) measure of sampling adequacy and Bartlett’s test of sphericity were used to evaluate the suitability of the data for factor analysis. Second, confirmatory factor analysis (CFA) was performed in AMOS (version 26) using the maximum likelihood estimation method to assess construct validity, including both convergent and discriminant validity. The evaluation of model validity was based on multiple criteria, including standardized factor loadings, composite reliability (CR), and average variance extracted (AVE). Third, SEM was employed to test the hypothesized structural model, estimating the relationships among latent variables and assessing overall model fit. Model fit was evaluated using several indices: the goodness-of-fit index (GFI), normed fit index (NFI), comparative fit index (CFI), incremental fit index (IFI), Tucker–Lewis index (TLI), and root mean square error of approximation (RMSEA).

To ensure the adequacy of the sample size for SEM, prior research suggests a minimum of 100 to 150 participants (Kline, 2010). With a final sample of 257 participants, the dataset exceeds the recommended threshold, supporting the robustness of the SEM analysis.

## 4. Results

### 4.1. Measurement Validation

To evaluate the instrument validity and reliability, we used Cronbach α to assess internal reliability, the KMO to examine whether the analyses yielded distinct and reliable factors, CR to assess the construct reliability, and AVE to assess convergent and discriminant validity criteria using confirmatory factor analysis (Hair et al., 2014). Table 2 presents the instrument’s validity and reliability. As shown, the internal reliability using Cronbach’s α indicates that all the items are from 0.68 to 0.83, indicating good reliability. All KMO values for individual items were above 0.70, which is well above the limit of 0.5. The CR for each construct ranged from 0.76 to 0.91, all higher than the recommended threshold of 0.60. The AVE of each construct ranged from 0.51 to 0.74 and exceeded the cut-off value of 0.5, demonstrating adequate construct validity because more than 50% variance is explained by the construct. The results indicate satisfactory internal reliability and convergent and discriminant validity.

### 4.2. Test of Structural Model

**Figure 2:**
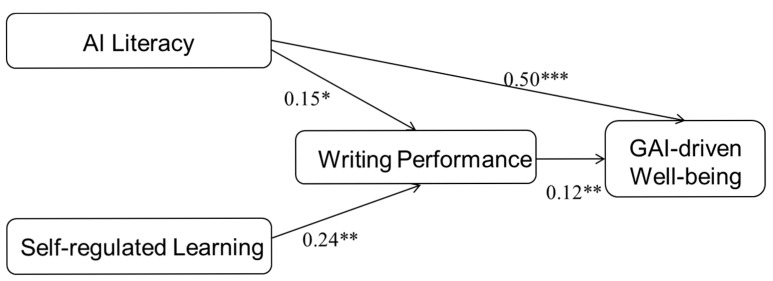
Structural equation model analysis. Note. * *p* < 0.05, ** *p* < 0.01, *** *p* < 0.001.

To evaluate the structural model, we examined both the overall model fit and the statistical significance of the hypothesized paths among the four core variables. The model demonstrated acceptable fit to the data, as indicated by the following indices: χ²/df = 2.67, GFI = 0.89, NFI = 0.86, CFI = 0.91, IFI = 0.91, TLI = 0.88, and RMSEA = 0.08. SEM was used to test the hypothesized relationships, including the direct effects of AI literacy and self-regulated learning on writing performance and the direct effects of AI literacy and writing performance on GAI-driven well-being. Figure 2 presents the structural model with standardized path coefficients, and Table 3 summarizes the path estimates.

As Table 3 shows, the path analysis revealed that both AI literacy and SRL were positively associated with writing performance, with SRL being the stronger predictor. AI literacy (β = 0.153, *p* < 0.05) and self-regulated learning (β = 0.237, *p* < 0.01) were significantly linked to writing performance. Furthermore, AI literacy was positively associated with GAI-driven well-being (β = 0.503, *p* < 0.001), indicating that learners with higher AI literacy tended to report higher digital well-being in GAI-supported environments. Writing performance was also positively associated with GAI-driven well-being (β = 0.120, *p* < 0.01). These results support the hypothesized model and demonstrate that both AI literacy and SRL strategies are important correlates of students’ writing performance and well-being in the context of GAI-assisted learning.

### 4.3. Exploratory Mediation Analysis

To further explore the underlying mechanisms linking learner competencies and well-being in GAI-supported environments, we conducted a mediation analysis to examine whether writing performance statistically mediates the effects of AI literacy and SRL on GAI-driven well-being. In this model, AI literacy and SRL were modeled as independent variables, writing performance as the mediating variable, and GAI-driven well-being as the dependent outcome.

As shown in Table 4, in terms of indirect effects, the pathway from AI literacy to GAI-driven well-being through writing performance yielded a small but significant effect (β = 0.018). Similarly, SRL showed an indirect association with GAI-driven well-being through writing performance (β = 0.029). These findings suggest that writing performance acts as a partial mediator in the relationship between AI literacy and GAI-driven well-being and as a full mediator in the relationship between SRL and GAI-driven well-being.

The standardized total effects highlight the relative contribution of each variable to GAI-driven well-being. AI literacy emerged as the most influential predictor (β = 0.521), followed by writing performance (β = 0.120) and SRL (β = 0.029). These results underscore the central role of AI literacy, not only in its direct association with GAI-driven well-being but also in indirect linkage through academic performance. Furthermore, the findings suggest that while SRL may not directly affect GAI-driven well-being, it remains a critical antecedent of performance-related gains, which in turn is associated with better well-being outcomes in GAI-mediated learning contexts.

## 5. Discussion

This study employed SEM to investigate the factors associated with students’ writing performance and GAI-driven well-being in the context of GAI-supported academic writing. The findings provide empirical support to key assumptions derived from existing theories and extend the application of relevant theoretical frameworks in the domain of GAI-supported learning.

### 5.1. The Impact of AI Literacy and SRL on Writing Performance

The results demonstrated that both AI literacy and SRL were significantly and positively associated with writing performance, with SRL exerting a stronger effect. This finding aligns with previous studies on GAI-supported writing. For instance, J. Kim et al. (2025) found that students with high AI literacy perceived themselves as more capable of crafting precise language and generating relevant content, thereby receiving outputs that were both accurate and responsive to their academic needs. These results highlight the potential value of AI integration in academic writing. Moreover, the findings are consistent with SRL theory (Zimmerman, 2002), which posits that students with higher self-regulatory capacities are more adept at employing metacognitive strategies and adjusting their learning behaviors to improve performance. Pintrich (2000) further emphasized that SRL enables learners to actively monitor and control their cognitive engagement, which is particularly important in complex tasks such as academic writing.

### 5.2. The Impact of AI Literacy on GAI-Driven Well-Being

The findings also revealed that AI literacy was positively associated not only with writing performance but also with students’ GAI-driven well-being. In recent years, the role of AI in enhancing students’ learning experiences has received increasing attention. For example, Luckin et al. (2016) noted that AI technologies can improve engagement and satisfaction by offering personalized learning paths, real-time feedback, and intelligent recommendation systems. M. P.-C. Lin and Chang (2020) similarly reported that AI support improves writing structure and contributes to a more enjoyable learning experience. Furthermore, J. Kim and Cho (2023) found that students viewed AI tools as collaborative partners—helping them brainstorm ideas, foster creativity, and reduce feelings of isolation during writing tasks. The results of the present study further suggest that students who are proficient in using AI tools not only benefit from enhanced writing quality and reduced writing anxiety but also tend to experience a stronger sense of control over the learning process. These findings carry important practical implications for promoting AI literacy as a key competence in higher education.

### 5.3. The Mediating Role of Writing Performance in the Relationship Between AI Literacy, SRL, and GAI-Driven Well-Being

This study further identified writing performance as a potential mediating variable linking AI literacy and SRL to GAI-driven well-being. This finding highlights the dynamic interplay between technological competence, learning strategies, and psychological well-being. The results are consistent with the Control-Value Theory of Achievement Emotions (Pekrun, 2006; Pekrun et al., 2014), which posits that students experience more positive achievement emotions when they perceive high levels of control (an individual’s perception of his or her ability to influence the achievement of activities and outcomes) over their learning processes (e.g., through SRL). These positive emotions, in turn, are expected to relate to better academic outcomes across a variety of learning tasks. In this study, writing performance served as a statistical link through which the benefits of AI literacy and SRL were translated into enhanced well-being in GAI-supported environments.

This mediating effect supports a systems perspective on learner development, suggesting that emotional, cognitive, and behavioral dimensions must be considered collectively in order to understand how students adapt and succeed in GAI-supported learning contexts.

## 6. Conclusions, Limitations, and Implications

### 6.1. Conclusions

In response to the growing integration of GAI into higher education, this study examined how AI literacy and SRL relate to students’ writing performance and GAI-driven digital well-being in academic writing contexts. Drawing upon a sample of 257 university students in China and employing SEM, this study identified the significant associations among AI literacy, SRL, writing performance, and GAI-driven well-being. Writing performance was found to function as a potential statistical mediator between learner competencies and well-being, suggesting a central role in the overall pattern of relationships in GAI-supported academic contexts.

This study makes several important theoretical contributions to the literature on GAI-supported education and student development: First, by integrating AI literacy and SRL into a unified analytical framework, this study advances understanding of how cognitive and behavioral competencies are jointly associated with academic performance and well-being in GAI-supported learning. The model moves beyond prior research that treated these variables in isolation, offering empirical evidence of their combined associations on both writing performance and GAI-driven well-being. Second, the research extends the application of the Self-Regulated Learning theory (Zimmerman, 2002) to the context of GAI-mediated academic writing, demonstrating how learners’ perceptions and use of GAI tools may be linked to task outcomes and psychological experiences. Third, by uncovering the mediating role of writing performance between learner traits and well-being, this study offers a novel contribution to the Control-Value Theory of Achievement Emotions (Pekrun, 2006), suggesting that students’ perceived control and value in GAI-supported learning environments can enhance positive emotions and academic flourishing.

### 6.2. Limitations

Despite its contributions, this study has several limitations. First, the model did not include potential control variables such as prior academic achievement, baseline digital literacy, access to GAI tools, and socioeconomic status. The omission of these factors may introduce bias and limit the precision of the estimated relationships. Second, the use of cross-sectional survey data restricts causal inference. Although the mediation model was theoretically informed, the findings reflect correlational patterns rather than confirmed temporal or causal effects. Future research should adopt longitudinal or experimental designs to validate the proposed pathways. Third, this study focused on students in Chinese universities, which may limit the generalizability of the results to other cultural and educational contexts. Further cross-cultural studies are needed to explore whether these findings hold in diverse settings.

### 6.3. Implications

This study underscores the significant associations between AI literacy, SRL, and both academic performance and well-being in GAI-supported education. As AI transforms learning and writing, institutions must cultivate not only technical skills but also students’ capacities for reflection, regulation, and responsible use.

First, universities should offer foundational courses (e.g., AI Literacy Foundations) that integrate conceptual knowledge, real-world applications, and ethical considerations. These should be taught by qualified instructors and include case-based analysis of model risks and limitations. In parallel, institutions should develop formative, process-based assessments to encourage effective and accountable use of GAI tools. Second, instructional design should incorporate SRL practices through goal setting, self-monitoring, and reflection. Instructors can require students to maintain writing logs that record their use of GAI tools and reflect on their learning. These reflections enable teachers to provide targeted feedback and support SRL growth. Third, writing should be seen not only as an output but as a developmental process supporting cognitive and emotional engagement. Educators should scaffold writing tasks that enhance confidence and promote student agency. Finally, institutions should establish ethical guidelines and supportive digital ecosystems to ensure the sustainable use of GAI in education—addressing long-term impacts on students’ emotional balance, social interaction, and academic integrity.

## Figures and Tables

**Table 1:** Distribution of participants by gender, grade level, nationality, and academic discipline.

Demographic Factors	Category	n	%
Gender	Male	89	34.6%
	Female	168	65.3%
Grade level	Freshmen	12	4.6%
	Sophomores	47	18.2%
	Juniors	74	28.7%
	Seniors	51	19.8%
	1st-year graduate	43	16.7%
	2nd-year graduate	24	9.3%
	3rd-year graduate	6	2.3%
Nationality	China	224	87.1%
	Other countries	33	12.8%
Academic discipline	Humanities and arts	76	29.5%
	Social sciences	130	50.5%
	Natural sciences	28	10.8%
	Engineering sciences	23	8.9%

**Table 2:** Instrument validity and reliability.

Measure	Number of Items	Cronbach’s α	KMO	CR	AVE
AI literacy	5	0.83	0.83	0.85	0.54
Self-regulated learning	3	0.75	0.70	0.76	0.51
Writing performance	4	0.68	0.70	0.83	0.57
GAI-driven well-being	4	0.82	0.75	0.91	0.74

**Table 3:** Path coefficients and significance levels.

Path	Standardized Coefficient (*β*)	SE	t
AI literacy→writing performance	0.153	0.026	1.982 *
Self-regulated learning→writing performance	0.237	0.041	2.642 **
AI literacy→GAI-driven well-being	0.503	0.095	5.131 ***
Writing performance→GAI-driven well-being	0.120	0.129	2.657 **

Note. * *p* < 0.05, ** *p* < 0.01, *** *p* < 0.001.

**Table 4:** Results of exploratory mediation analysis for GAI-driven well-being: direct effect, indirect effect, and total effect.

		Independent Variables	AI Literacy	Self-Regulated Learning	Writing Performance
	Dependent Variables				
Standardized Direct effects	Writing performance		0.153	0.237	—
	GAI-driven well-being		0.503	—	0.120
Standardized Indirect effects	GAI-driven well-being		0.018	0.029	—
Standardized Total effects	Writing performance		0.153	0.237	—
	GAI-driven well-being		0.521	0.029	0.120

## Data Availability

According to the Non-Disclosure Agreement (NDA), the data cannot be disclosed through public channels. The corresponding author can provide the datasets used and/or analyzed during the current study upon reasonable request.

## Appendix A. Survey Items Used in This Study

All items were measured on a 5-point Likert scale ranging from 1 (strongly disagree) to 5 (strongly agree).

1. AI Literacy

(1) I am able to prompt AI tools accurately by inputting text, images, or other forms of information to complete specific tasks.

(2) I can select and apply appropriate AI skills or functionalities based on my individual needs.

(3) I am proficient in using suitable AI tools to solve practical problems in different task scenarios.

(4) I demonstrate creativity when using AI and can integrate multiple tools to meet specific requirements.

(5) I am capable of combining AI knowledge with my academic or professional domain to generate new ideas and outcomes.

2. Self-Regulated Learning (SRL)

(1) I am able to resolve difficulties that arise during the learning process.

(2) I make plans to manage my study time effectively.

(3) I actively reflect on and summarize my learning experiences.

3. Writing Performance

(1) I use GAI to generate inspiration and ideas for my writing.

(2) I have improved my vocabulary usage through the support of GAI.

(3) I have enhanced my grammatical accuracy through the use of GAI.

(4) I have improved my overall language expression with the help of GAI.

4. GAI-Driven Well-Being

(1) When using GAI, I am able to recognize its value in supporting my learning and daily life.

(2) GAI has helped me improve the quality of my writing, such as clarity and coherence.

(3) Using GAI has reduced my anxiety related to Chinese writing.

(4) I feel satisfied with the overall experience of using GAI in support of my Chinese writing.
